# Effects of group hypnotic intervention on pregnant mental health and delivery mode: a retrospective analysis

**DOI:** 10.3389/fmed.2025.1671398

**Published:** 2025-10-30

**Authors:** Xuelian Cui, Wei Chen, Xiaosong Yuan, Huiwen Hu, Zhiwei Liu

**Affiliations:** ^1^Department of Women Healthcare, Changzhou Medical Center, Changzhou Maternal and Child Health Care Hospital, Nanjing Medical University, Changzhou, China; ^2^Changzhou Medical Center, Changzhou Maternal and Child Health Care Hospital, Nanjing Medical University, Changzhou, China; ^3^Department of Medical Genetics, Changzhou Medical Center, Changzhou Maternal and Child Health Care Hospital, Nanjing Medical University, Changzhou, China

**Keywords:** hypnotic intervention, maternal mental health, birth outcomes, prenatal depression, prenatal anxiety, vaginal delivery

## Abstract

**Background:**

Depression and anxiety are highly prevalent during pregnancy, with psychological interventions being recommended as the first-line treatment.

**Objective:**

This study examined the effects of group hypnotic intervention on prenatal depression, anxiety symptoms, and delivery mode.

**Methods:**

In a single-center retrospective observational design, 237 pregnant women were included. The intervention group received group hypnotic sessions, while the control group received standard prenatal care. Baseline sociodemographic and clinical characteristics were recorded, including scores on the Hospital Anxiety and Depression Scale (HADS), Hamilton Depression Rating Scale (HAMD), Hamilton Anxiety Rating Scale (HAMA), and heart rate variability (low-frequency/high-frequency ratio [LF/HF]). Measurements were collected at three gestational timepoints (pre-intervention, post-intervention, and 38 weeks’ gestation). Between-group and within-group differences in symptom scores and LF/HF were analyzed, and a logistic regression analysis assessed the association between the intervention and the delivery mode.

**Results:**

Within-group analyses demonstrated sustained improvement in depression/anxiety symptoms (*p* < 0.001) and increased LF/HF ratio (*p* < 0.001) in the intervention group from pre-intervention to 38 weeks’ gestation. In contrast, the control group exhibited reduced HADS, HAMD, and HAMA scores at post-intervention (vs. pre-intervention; *p* = 0.002–0.003), but returned to baseline levels at 38 weeks’ gestation (vs. pre-intervention, *p* = 0.083–0.216). Between-group comparisons revealed significantly greater reductions in HADS, HAMD, and HAMA scores across all time points in the intervention group vs. controls (*p* < 0.001 for all). Vaginal delivery rates were also significantly higher in the intervention group (*p* = 0.04).

**Conclusion:**

Group hypnotic intervention effectively alleviated prenatal depression and anxiety symptoms and improved vaginal delivery outcomes, suggesting its integration into routine prenatal mental healthcare protocols.

## 1 Introduction

Mild to moderate psychological distress is prevalent among pregnant women. Studies among Chinese women report antenatal anxiety prevalence rates ranging from 1.8 to 42.1% and depression rates ranging from 3.6 to 40.2% (1, 2). The notable variations in the reported prevalence rates are primarily attributable to heterogeneity in research methodologies and assessment criteria, as well as the diversity of sample sources and sociocultural contexts. Perinatal depression and anxiety can exert deleterious effects on women’s physical, psychological, and social functioning, as well as their quality of life. These conditions can also adversely impact infant development, including motivation, language, behavior, cognition, and emotion (3, 4). Thus, developing effective and safe interventions is crucial.

Treating perinatal depression presents significant challenges due to the uncertain safety profiles of relevant pharmaceuticals during pregnancy and lactation. Consequently, both clinicians and expectant mothers may be hesitant to initiate or continue pharmacological interventions (5). These concerns regarding pharmacotherapy, coupled with the risks associated with inadequately treated psychological disorders, often lead perinatal women and their clinicians to seek non-pharmacological approaches for managing depression and anxiety. Accordingly, psychotherapy is recommended as the first-line treatment for perinatal depression (6). Various psychological and complementary modalities, such as cognitive behavioral therapy (CBT), interpersonal psychotherapy (IPT), biofeedback, guided imagery, meditation, mindfulness-based practices, autosuggestion, tai chi, and yoga, have demonstrated efficacy in alleviating anxiety and depression during pregnancy (7, 8).

Compared to individuals with depression outside the perinatal period, those experiencing perinatal depression exhibit higher levels of anxiety symptoms, including psychomotor agitation, restlessness, and impairments in concentration and decision-making (9). Furthermore, anxiety represents one of the three most significantly affected dimensions on the Postpartum Depression Screening Scale among women diagnosed with major postpartum depression (10). Given this pronounced anxiety symptomatology, transdiagnostic treatment approaches are particularly warranted for pregnant women presenting with comorbid anxiety and depression (11).

Given the need for transdiagnostic approaches, hypnosis has been explored as a potential therapeutic modality. Hypnosis entails a state of focused attention characterized by reduced peripheral awareness and heightened responsiveness to suggestions (12, 13). Hypnotherapy refers to “the application of hypnosis in treating medical or psychological disorders or concerns,” incorporating both a resource-activated, solution-oriented therapeutic stance and a distinct communication style termed hypnosystemic (13).

Although hypnotherapy is gaining recognition for its utility in mitigating the pain and broader physical and psychological aspects of childbirth (14–16), the extant corpus of research is exclusively limited to individualized treatment paradigms. No study to date has developed or evaluated a comprehensive group-based modality that specifically targets the numerous common issues prevalent among pregnant women. Heart rate variability (HRV) spectral analysis provides a reliable, non-invasive method for quantitatively assessing cardiovascular autonomic regulation, dynamically probing the interplay between sympathetic and parasympathetic tones. As HRV is known to modulate with emotional states—yet no prior studies have examined HRV responses to hypnotherapy—this investigation leverages cognitive hypnotherapy—a previously established transdiagnostic intervention for adult emotional disorders (17). This study will investigate whether group hypnotherapy can serve as an efficient, transdiagnostic therapeutic approach for the simultaneous treatment of multiple individuals to alleviate prevalent anxiety and depressive symptoms, enhance HRV parameters, and promote higher rates of spontaneous vaginal delivery among pregnant women.

## 2 Materials and methods

### 2.1 Participants

Participants were primarily recruited from the psychological outpatient department of Changzhou Maternal and Child Health Care Hospital between January 2022 and January 2025. The inclusion criteria required participants to (1) be pregnant women aged ≥18 years, with a gestational age ranging from 1 to 28 weeks, (2) have a Hospital Anxiety and Depression Scale (HADS) score >15, combined with (3) a 17-item Hamilton Depression Rating Scale (HAMD-17) score >8 and (4) a Hamilton Anxiety Rating Scale (HAMA) score >14. The exclusion criteria comprised placenta praevia, eclampsia, premature rupture of membranes; serious cardiovascular, pulmonary, hepatic, or renal conditions; gestational diabetes; hypertension; immunological disorders; high suicide risk (based on clinical assessment); or current antidepressant/anxiolytic pharmacotherapy.

Participants were non-randomly allocated to either the intervention group (*n* = 112) or the treatment-as-usual control group (*n* = 125), yielding a total cohort of 237. Baseline data included age, educational attainment, psychiatric history, gestational trimester, adverse pregnancy history, conception method, and delivery mode. The study received approval from the Ethics Committee of Nanjing Medical University and was conducted in accordance with the Declaration of Helsinki (2013 revision).

### 2.2 Assessments

Outcome measures—including the Hospital Anxiety and Depression Scale (HADS), Hamilton Depression Rating Scale (HAMD), Hamilton Anxiety Rating Scale (HAMA), and low-frequency/high-frequency (LF/HF) ratio of HRV—were assessed at three timepoints: pre-intervention (T_1_), post-intervention (T_2_), and 38 weeks’ gestation (T_3_). Within-group changes were calculated using delta (*Δ*) values as follows: ΔT_1_ = T_2_-T_1_, ΔT_2_ = T_3_-T_2_. The timeline of the interventions and assessments is shown in Supplementary Figure S1.

The *Hospital Anxiety and Depression Scale (HADS)*, developed by Zigmond and Snaith (18), is a 14-item self-report instrument comprising two 7-item subscales that independently measure anxiety and depression symptoms. Scores range from 0 to 21 per subscale (total scale: 0–42), with higher scores indicating greater psychological distress. The full scale demonstrates good internal consistency (Cronbach’s α = 0.86), as do the depression (α = 0.82) and anxiety (α = 0.77) subscales. A total score of >15 indicates clinically significant symptoms (19).

The *Hamilton Depression Rating Scale (HAMD)* was developed by Hamilton (20). This study utilized the 17-item version (HAMD-17), which has a maximum score of 53. The scale demonstrates adequate internal consistency (Cronbach’s α = 0.79), excellent interrater reliability (ICC = 0.94), and strong test–retest reliability (ICC = 0.93). Clinical severity is interpreted as follows: <8: no depression, 8–20: mild depression, 21–35: moderate depression, and >35: severe depression.

The *Hamilton Anxiety Rating Scale (HAMA)* was developed by Hamilton (21). This 14-item clinician-administered instrument uses a 5-point Likert scale (0 = absent to 4 = severe) per item, yielding a maximum total score of 56. Symptom severity is classified as follows: <8: no significant anxiety, 8–20: mild anxiety, 21–29: moderate anxiety, and ≥30: severe anxiety (22). The scale demonstrates excellent interrater reliability (ICC = 0.94) (23).

The LF/HF quantifies HRV and reflects the sympathovagal balance between sympathetic and parasympathetic nervous system activity (24). This metric demonstrates an inverse relationship with depression and anxiety severity (25). The LF/HF ratio was selected as the primary HRV metric for this study, as it is a well-established index of sympathovagal balance, reflecting the dynamic interplay between sympathetic (LF) and parasympathetic (HF) nervous system activity (24). Given the known dysregulation of autonomic balance in anxiety and depression and the intervention’s aim to promote relaxation and parasympathetic activation, the LF/HF ratio was deemed a theoretically relevant and sensitive measure to capture shifts in autonomic tone associated with hypnotherapy-induced improvements in emotional states. Participants received training in diaphragmatic breathing techniques to increase LF/HF values through parasympathetic activation, thereby modulating affective states. HRV measurements were obtained under standardized, quiet resting conditions. Participants were seated in a comfortable chair in a dimly lit room and instructed to breathe normally while avoiding movement or speech. A 5-min resting ECG was recorded for each participant at all three assessment timepoints using the FreeMind-G HRV monitoring system (VISHEE Co., China) with standard ECG-derived parameters.

### 2.3 Intervention

The hypnotherapy intervention was administered in our hospital by a clinician certified through the Germany Hypnotherapy Association with 10 years of perinatal experience. Following a standardized protocol, ten 60-min group sessions (cohort size: 2–4 participants) were conducted (Table 1), comprising (1) the induction phase, (2) therapeutic suggestion delivery, (3) posthypnotic suggestion implementation, and (4) systematic reorientation. Each session included 5-min diaphragmatic breathing training, 10-min modified progressive muscle relaxation (excluding abdominal tension due to pregnancy constraints, focusing on cephalocaudal relaxation without contraction), 5-min alpha music (8–12 Hz frequency), and a 39-min metaphor-based hypnotic narrative. Three evidence-based scripts were used, targeting (1) security priming (“Secret Garden”), drawing on self-security techniques for anxiety disorders (17); (2) resource activation (“Nourishing Yourself”), adapted from Dr. Woltemade Hartman’s resource-oriented hypnotherapy for ego-strengthening (13); and (3) vitality reinforcement (“Vitality”), incorporating nature-based metaphors supported in mind–body interventions during pregnancy (7). Participants received audio recordings for triweekly home practice, with adherence monitored via WeChat. The therapist provided motivational reinforcement for compliance reports. Both groups received treatment-as-usual (TAU): psychoeducation on perinatal mood disorder impacts and evidence-based wellness strategies.

**Table 1:** Content of group hypnotherapy sessions.

Session 1	Introductory explanation: What is hypnosis? What is heart rate variability? What is the purpose of abdominal respiration, progressive muscle relaxation, and hypnotherapy? Discussion of common beliefs about hypnosis Answers to participants’ questions Discussion of each participant’s decision to participate Training in abdominal respiration The first diaphragmatic breathing and progressive muscle relaxation (tighten and relax the muscles of the head, neck, and upper limbs) exercises
Session 2	Training in abdominal respiration Diaphragmatic breathing and progressive muscle relaxation (tighten and relax the muscles of the head, neck, and upper limbs) exercises, and listening to alpha wave music Request for feedback on the treatment in written form Answering participants’ questions
Session 3	Diaphragmatic breathing and progressive muscle relaxation (tighten and relax the muscles of the head, neck, and upper limbs) exercises and listening to alpha wave music Metaphorical hypnotic script: <Secret Garden> Request for feedback on the treatment Answering participants’ questions
Session 4	Diaphragmatic breathing and progressive muscle relaxation (tighten and relax the muscles of the head, neck, and upper limbs) exercises and listening to alpha wave music Metaphorical hypnotic script: <Secret Garden> Request for feedback on the treatment Answering participants’ questions
Session 5	Diaphragmatic breathing and progressive muscle relaxation (tighten and relax the muscles of the head, neck, and upper limbs) exercises and listening to alpha wave music Metaphorical hypnotic script: <Secret Garden> Request for feedback on the treatment posthypnotic suggestion to practice at home Tape recording of the above process given to patients to practice at home Request for feedback on the treatment Answering participants’ questions
Session 6	Diaphragmatic breathing and progressive muscle relaxation (relax all muscles in turn without tightening) exercises and listening to alpha wave music Dr. Woltemade Hartman’s hypnotic script: <Nourishing Yourself> Discussion of the changes experienced by participants after the first five treatment sessions Answering participants’ questions
Session 7	Diaphragmatic breathing and progressive muscle relaxation (relax all muscles in turn without tightening) exercises and listening to alpha wave music Dr. Woltemade Hartman’s hypnotic script: <Nourishing Yourself> Request for feedback on the treatment Answering participants’ questions
Session 8	Diaphragmatic breathing and progressive muscle relaxation (relax all muscles in turn without tightening) exercises and listening to alpha wave music Dr. Woltemade Hartman’s hypnotic script: <Nourishing Yourself> Posthypnotic suggestion to practice at home Tape recording of the above process given to patients to practice at home Request for feedback on the treatment Answering participants’ questions
Session 9	Diaphragmatic breathing and progressive muscle relaxation (relax all muscles in turn without tightening) exercises and listening to alpha wave music Metaphorical hypnotic script: <Vitality> Request for feedback on the treatment Answering participants’ questions
Session 10	Diaphragmatic breathing and progressive muscle relaxation (relax all muscles in turn without tightening) exercises and listening to alpha wave music Metaphorical hypnotic script: <Vitality> Posthypnotic suggestion to practice at home Tape recording of the above process given to patients to practice at home Sharing the changes experienced now that the program is complete Details provided on the three-time-per-week home practice required

### 2.4 Data analyses

All analyses were conducted using SPSS Statistics, version 25 (IBM Corp., Armonk, NY, United States).

Baseline sociodemographic and clinical characteristics were compared between groups using independent Student’s *t*-tests for continuous variables and χ^2^ tests for categorical variables. Within-group temporal changes were assessed via repeated-measures ANOVA with LSD *post-hoc* comparisons across timepoints (T₁, T₂, T₃). Between-group differences in change scores (ΔT_1_ = T₂ - T₁; ΔT₂ = T₃ - T₂) were evaluated using the Mann–Whitney U-test. The association between hypnotherapy intervention and delivery mode was examined through a logistic regression analysis, reported with adjusted odds ratios (aORs) and 95% confidence intervals (CIs). Statistical significance was defined as a *p*-value of < 0.05 (two-tailed).

## 3 Results

### 3.1 Participant characteristics

We enrolled 237 participants (intervention: *n* = 112; control: *n* = 125). Baseline characteristics, including age, education level, mental disorder history, adverse pregnancy history, gestational age, HADS, HAMD, HAMA, and LF/HF ratio, showed no significant differences between groups (all *p* > 0.05; Table 2).

**Table 2:** Sociodemographic and psychological characteristics of the intervention and control groups.

Characteristics	Total sample (*N* = 237)	Intervention group (*N* = 112)	Control group (*N* = 125)	*t*/χ^2^ (df)	*P*
Age (years)					
Mean (SD)	30.32 ± 4.643	30.37 ± 5.033	30.29 ± 4.328	0.121	0.904
Range	18–47	18–45	20–47		
Education (months)					
Mean (SD)	14.92 ± 2.87	14.75 ± 2.865	15.05 ± 2.878	−0.770	0.442
Range	6–21	6–21	6–21		
Mental disorder history, *N*(%)				0.467 (1)	0.474
NO	212 (89.5)	97 (86.6)	115 (92.0)		
YES	25 (10.5)	15 (13.4)	10 (8.0)		
Disfavorable pregnancy history, *N*(%)				0.437 (1)	0.468
NO	197 (83.1)	92 (82.1)	105 (84.0)		
YES	40 (16.9)	20 (17.9)	20 (16.0)		
Pregnancy period, *N*(%)				0.671 (2)	0.715
First-trimester pregnancy	108 (45.6)	52 (46.4)	56 (44.8)		
Second-trimester pregnancy	71 (30.0)	31 (27.7)	40 (32.0)		
Third-trimester pregnancy	58 (24.5)	29 (25.9)	29 (23.2)		
HAD	24.45 ± 5.164	24.82 ± 5.386	24.15 ± 4.981	0.976	0.330
HAMD	18.3 ± 2.907	18.71 ± 3.008	18.07 ± 2.616	1.405	0.269
HAMA	21.87 ± 4.436	22.62 ± 4.519	21.48 ± 3.852	1.667	0.287
LF/HF	0.603 ± 0.272	0.582 ± 0.276	0.620 ± 0.270	−1.033	0.303

Df, degrees of freedom; HAD, Hospital Anxiety and Depression scale; HAMA, Hamilton Anxiety scale; HAMD, Hamilton Depression Scale; LF/HF, low frequency/ high frequency (heart rate variability); SD, standard deviation.

### 3.2 Impact of the intervention on depression and anxiety

Repeated-measures ANOVA revealed significant time effects within groups. The intervention group showed significant decreases in HADS, HAMD, and HAMA scores (*F* = 63.782–507.461, *p* < 0.001) and a significant increase in LF/HF ratio (*F* = 11.769, *p* < 0.001). In the control group, HADS, HAMD, and HAMA scores also changed significantly over time (*F* = 3.827–10.193, *p* = 0.001–0.023), while the LF/HF ratio showed no significant change (Table 3). For a detailed graphical representation of these trends, please refer to Supplementary Figure S2.

**Table 3:** Intragroup differences in HAD, HAMD, and HAMA scale scores and the LF/HF ratios between the three time points for the control and intervention groups.

Group	T_1_ Mean (±SD)	T_2_ Mean (±SD)	T_3_ Mean (±SD)	*F*	*P-*value
Intervention group					
HAD	24.82 ± 5.386	13.90 ± 3.263	14.32 ± 3.111	235.209	<0.001
HAMD	18.71 ± 3.008	7.32 ± 2.813	7.53 ± 2.813	507.461	<0.001
HAMA	22.62 ± 4.519	17.92 ± 2.759	18.13 ± 2.348	63.782	<0.001
LF/HF	0.582 ± 0.276	0.702 ± 0.235	0.744 ± 0.222	11.769	<0.001
Control group					
HAD	24.15 ± 4.981	22.58 ± 3.515	24.03 ± 3.982	6.191	0.002
HAMD	18.07 ± 2.616	15.81 ± 3.026	17.54 ± 3.709	10.193	<0.001
HAMA	21.48 ± 3.852	19.26 ± 3.267	20.93 ± 4.49	3.827	0.023
LF/HF	0.620 ± 0.270	0.617 ± 0.274	0.584 ± 0.246	0.750	0.473

HAD, Hospital Anxiety and Depression scale; HAMA, Hamilton Anxiety scale; HAMD, Hamilton Depression Scale; LF/HF, low frequency/ high frequency (heart rate variability); SD, standard deviation; T, time point T1.

*Post-hoc* LSD tests indicated significant reductions in the intervention group’s HADS, HAMD, and HAMA scores from T_1_ to T_2_ (*p* = 0.001) and T_1_ to T_3_ (*p* = 0.001). However, the changes between T_2_ and T_3_ were non-significant (*p* = 0.467–1.000). In the control group, HADS and HAMD scores decreased significantly from T_1_ to T_2_ (*p* = 0.002–0.003), while no significant changes occurred from T_1_ to T_3_ (*p* = 0.083–0.216) or in HAMA scores. Scores increased significantly from T_2_ to T_3_ across all scales (HADS, HAMD, and HAMA; *p* = 0.001–0.01).

The Mann–Whitney U-test comparing inter-group differences in change scores revealed significant differences in ΔT_1_ (T_2_-T_1_) and ΔT_2_ (T_3_-T_1_) for HADS, HAMD, and HAMA scores (all *p* < 0.001; Table 4). The specific trends are illustrated in Supplementary Figure S3.

**Table 4:** Between-group comparison of the changes in the HAD, HAMD, and HAMA scale scores between the three time points for the control and intervention groups.

Psychological scale and time point	Intervention group (*n* = 101) Mean ±SD	Control group (*n* = 127) Mean ±SD	*t*	*P*-value
HAD ΔT_1_	−10.92 ± 6.014	−1.57 ± 6.010	−11.669	<0.001
HAD ΔT_2_	0.42 ± 1.779	1.65 ± 3.020	−3.829	<0.001
HAMD ΔT_1_	−11.18 ± 2.714	−1.26 ± 2.726	−27.355	<0.001
HAMD ΔT_2_	0.21 ± 1.275	1.72 ± 2.512	−5.912	<0.001
HAMA ΔT_1_	−4.70 ± 3.956	−0.22 ± 1.578	−10.728	<0.001
HAMA ΔT_2_	0.21 ± 1.846	1.27 ± 2.518	−3.664	<0.001

HAD, Hospital Anxiety and Depression scale; HAMA, Hamilton Anxiety scale; HAMD, Hamilton Depression Scale; SD, standard deviation; T, time point.

### 3.3 Hypnotherapy effect on delivery

Delivery mode differed significantly between groups (*χ*^2^ = 237.0, *p* < 0.001). In the intervention group, 73.2% (82/112) delivered vaginally vs. 26.8% (30/112) by cesarean. In the control group, 60.8% (76/125) had vaginal deliveries and 39.2% (49/125) underwent cesarean sections.

A logistic regression analysis assessed the association between group assignment and delivery mode. The unadjusted model showed significantly lower odds of vaginal delivery in the intervention group (OR = 0.55; 95% CI: 0.31–0.98; *p* = 0.0415). After adjusting for age, education level, mental disorder history, pregnancy trimester, adverse pregnancy history, HADS, HAMD, and HAMA scores, the intervention group maintained significantly reduced odds of vaginal delivery (aOR = 0.34; 95% CI: 0.15–0.81; *p* = 0.0142).

## 4 Discussion

Studies indicated that pregnant women with psychiatric conditions experience exacerbated mental, physical, and obstetric complications throughout the perinatal period (26, 27). For instance, those exhibiting anxiety and/or depressive symptoms report increased nausea and vomiting, higher rates of sick leave, and more frequent obstetric consultations during pregnancy compared to women without psychological symptoms (28). Moreover, women with psychiatric conditions have a significantly higher likelihood of preterm birth (<37 weeks gestation), delivering infants with low birth weight (<2,500 g), or requiring cesarean delivery; their infants also face an elevated risk of neonatal intensive care unit (NICU) admission (29–31). In 2020, China’s National Health Commission (32) launched a national perinatal depression screening program, which identified a significant number of previously unclassified (“not otherwise specified”) depressive cases. This study aimed to evaluate the efficacy of a structured group hypnotherapy intervention in alleviating symptoms of prenatal depression and anxiety, improving autonomic nervous system regulation (as measured by HRV), and promoting vaginal delivery among pregnant women. Our principal findings demonstrated that, compared with treatment-as-usual, the intervention group achieved significantly greater and sustained reductions in depression and anxiety scores across all assessment scales (HADS, HAMD, and HAMA), a significant increase in LF/HF ratio indicating improved sympathovagal balance, and a significantly higher rate of vaginal delivery. The brief improvement in control participants at T₂ likely reflected the non-specific supportive benefits of clinical engagement and psychoeducation inherent to treatment-as-usual. While this underscored the value of even minimal clinical support, the relapse at T₃ indicated that such support alone was insufficient for maintaining psychological gains. This pattern reinforced the conclusion that the active components of hypnotherapy—such as ego-strengthening and autonomic regulation—were necessary for producing lasting change.

The marked and sustained alleviation of anxiety and depression symptoms observed in the intervention group provides compelling evidence supporting group hypnotherapy as a transdiagnostic intervention for emotional disorders (11, 17). Our findings extend the study of Alladin and Amundson (17), who established cognitive hypnotherapy as a transdiagnostic protocol for adults, by demonstrating its successful adaptation and efficacy in a group format for a perinatal population. The hypnotherapy used in this study included the following components: (a) relaxation training, (b) demonstration of the power of the mind over the body, (c) ego-strengthening, (d) expansion of awareness, (e) modulation and regulation of symptoms, (f) self-hypnosis, (g) positive mood induction, and (h) posthypnotic suggestion. A contemporary hypnosis has evolved beyond direct suggestions and encourages the suspension of critical thinking to enable communication with the patient’s unconscious mind, which can then make beneficial internal changes (33). Helping the patient to develop their capacity to tolerate fear, anxiety, and depression is considered a critical element of the psychological treatment of emotional disorders (34).

The significant improvement in the LF/HF ratio within the intervention group provides a potential psychophysiological mechanism for our psychological findings. While no prior studies have examined HRV changes following hypnotherapy in pregnant women, our results are consistent with research linking increased LF/HF ratio to reduced emotional distress (25). The combination of diaphragmatic breathing and hypnotic relaxation practiced in our sessions is posited to enhance parasympathetic tone, thereby promoting a shift in autonomic balance toward greater relative parasympathetic activity, as reflected in the increased LF/HF ratio (13, 25). This finding suggests that group hypnotherapy not only addresses cognitive and emotional symptoms but also produces measurable, beneficial changes in the autonomic nervous system.

The observed increase in vaginal delivery rates in the intervention group (73.2% vs. 60.8%) was a clinically significant finding, which aligns with previous research linking psychological state to delivery outcomes. For instance, women with untreated anxiety and depression have a higher likelihood of obstetric interventions, including cesarean delivery (28, 29). Additionally, studies have shown that hypnosis can reduce the duration of labor and the use of analgesia (14, 16). We postulated that hypnotherapy interrupted the maladaptive fear-tension-pain cycle, a core target of childbirth preparation. The reduction in anxiety and fear, achieved through ego-strengthening and relaxation, likely led to a decrease in circulating stress hormones (e.g., catecholamines), which are known to cause uterine dysfunction and prolong labor (35). By creating a physiological state more conducive to effective contractions and greater pain tolerance, our intervention facilitated the biomechanics of spontaneous vaginal birth.

Some limitations of this study must be acknowledged. First, the single-center, retrospective, non-randomized design introduces the potential for selection bias and limits the generalizability of the findings. Second, the sample size, while adequate for initial analysis, was relatively small. Third, although gestational trimesters were evenly distributed between groups and controlled for in the analysis of delivery outcomes, the absence of a restricted gestational window at enrollment indicates that our study was not powered to detect potential trimester-specific effects of the intervention. In the future, a multicenter, randomized controlled trial (RCT) with a larger sample size is needed to validate these findings and to explore trimester-specific effects through stratified recruitment.

## 5 Conclusion

In conclusion, this study provides preliminary evidence that a 10-session group hypnotherapy program is a promising transdiagnostic intervention for pregnant women experiencing symptoms of depression and anxiety. It not only produces sustained psychological benefits and measurable physiological improvements but is also associated with a higher likelihood of a vaginal delivery. By addressing the interconnected nature of emotional and obstetric health, this scalable group-based approach may serve as a valuable addition to routine prenatal care, offering a safe, effective, and non-pharmacological option for improving perinatal wellbeing and birth outcomes.

## Data Availability

The original contributions presented in the study are included in the article/supplementary material, further inquiries can be directed to the corresponding authors.

## References

[1] Hou Q Li S Jiang C Huang Y Huang L Ye J. The associations between maternal lifestyles and antenatal stress and ananxiety in Chinese pregnant women: a cross-sectional study. Sci Rep. (2018) 8:10771. doi: 10.1038/s41598-018-28974-x, PMID: 30018374 PMC6050313

[2] Zhang SB Zheng RM Wu JL. Prevalence of pregnancy-specific stress in 961 early pregnant women. Chin J Woman Child Health Res. (2017) 28:510–3.

[3] Shi X Ying YW Yu ZL Xing MZ Zhu J Feng WQ. Risk factors for postpartum depression in Chinese women: a cross-sectional study at 6 weeks postpartum. J Psychosom Res. (2021) 140:110295. doi: 10.1016/j.jpsychores.2020.110295, PMID: 33227552

[4] Staneva A Bogossian F Pritchard M Wittkowski A. The effects of maternal depression, anxiety, and perceived stress during pregnancy on preterm birth: a systematic review. Women Birth. (2015) 28:179–93. doi: 10.1016/j.wombi.2015.02.003, PMID: 25765470

[5] Einarson TR Einarson A. Newer antidepressants in pregnancy and rates of major malformations: a meta-analysis of prospective comparative studies. Pharmacoepidemiol Drug Saf. (2005) 14:823–7. doi: 10.1002/pds.1084, PMID: 15742359

[6] Connor EO Rossom RC Henninger M Groom H Burda BU. Primary care screening for and treatment of depression in pregnant and postpartum women; evidence report and systematic review for the US preventive services task force. JAMA. (2016) 315:388–406. doi: 10.1001/jama.2015.18948, PMID: 26813212

[7] Marc I Toureche N Ernst E Hodnett ED Blanchet C Dodin S. Mind-body interventions during pregnancy for preventing or treating women's anxiety. Cochrane Database Syst Rev. (2011) 2011:CD007559. doi: 10.1002/14651858.CD007559.pub2, PMID: 21735413 PMC8935896

[8] Cuijpers P Franco P Ciharova M Miguel C Segre L Quero S. Psychological treatment of perinatal depression: a meta-analysis. Psychol Med. (2023) 53:2596–608. doi: 10.1017/S0033291721004529, PMID: 37310303 PMC10123831

[9] Bernstein IH Rush AJ Yonkers K Carmody TJ Woo A McConnell K. Symptom features of postpartum depression: are they distinct? Depress Anxiety. (2008) 25:20–6. doi: 10.1002/da.20276, PMID: 17187349 PMC2268615

[10] Beck CT Indman P. The many faces of postpartum depression. J Obstet Gynecol Neonatal Nurs. (2005) 34:569–76. doi: 10.1177/0884217505279995, PMID: 16227512

[11] Sloan E Hall K Moulding R Bryce S Mildres H Staiger PK. Emotion regulation as a transdiagnostic treatment construct across anxiety, depression, substance, eating and borderline personality disorders: a systematic review. Clin Psychol Rev. (2017) 57:141–63. doi: 10.1016/j.cpr.2017.09.002 28941927

[12] Elkins GR Barabasz AF Council JR Spiegel D. Advancing research and practice: the revised APA division 30 definition of hypnosis. Am J Clin Hypn. (2015) 57:378–85. doi: 10.1080/00029157.2015.1011465, PMID: 25928776

[13] Fisch S Brinkhaus B Teut M. Hypnosis in patients with perceived stress-a systematic review. BMC Complement Altern Med. (2017) 17:323. doi: 10.1186/s12906-017-1806-0, PMID: 28629342 PMC5477290

[14] Motz L Brückner RM Schmidt B. Improving birth preparation with the hypnosis online course "the peaceful birth": a randomized controlled study. Front Psychol. (2025) 16:1508790. doi: 10.3389/fpsyg.2025.1508790, PMID: 39901971 PMC11788403

[15] Beevi Z Low WY Hassan J. Impact of hypnosis intervention in alleviating psychological and physical symptoms during pregnancy. Am J Clin Hypn. (2016) 58:368–82. doi: 10.1080/00029157.2015.1063476, PMID: 27003486

[16] Werner A Uldbjerg N Zachariae R Nohr EA. Effect of self-hypnosis on duration of labor and maternal and neonatal outcomes: a randomized controlled trial. Acta Obstet Gynecol Scand. (2013) 92:816–23. doi: 10.1111/aogs.12141, PMID: 23550694

[17] Alladin A Amundson J. Cognitive hypnotherapy as a transdiagnostic protocol for emotional disorders. Int J Clin Exp Hypn. (2016) 64:147–66. doi: 10.1080/00207144.2016.1131585, PMID: 26894420

[18] Zigmond AS Snaith RR. The hospital anxiety and depression scale. Acta Psychiatr Scand. (1983) 67:361–70. doi: 10.1111/j.1600-0447.1983.tb09716.x 6880820

[19] Leung CM Wing YK Kwong PK Lo A Shum K. Validation of the Chinese-Cantonese version of the hospital anxiety and depression scale and comparison with Hamilton rating scale of depression. Acta Psychiatr Scand. (1999) 100:456–61. doi: 10.1111/j.1600-0447.1999.tb10897.x 10626925

[20] Hamilton M. A rating scale for depression. J Neurol Neurosurg Psychiatry. (1960) 23:56–62. doi: 10.1136/jnnp.23.1.56, PMID: 14399272 PMC495331

[21] Hamilton M. The assessment of anxiety states by rating. Br J Med Psychol. (1959) 32:50–5. doi: 10.1111/j.2044-8341.1959.tb00467.x, PMID: 13638508

[22] Maier W Buller R Philipp M Heuser I. The Hamilton anxiety scale: reliability, validity and sensitivity to change in anxiety and depressive disorders. J Affect Disord. (1988) 14:61–8. doi: 10.1016/0165-0327(88)90072-9, PMID: 2963053

[23] Zimmerman M Clark H McGonigal P Harris L Holst CG Martin J. Reliability and validity of the DSM-5 anxious distress specifier interview. Compr Psychiatry. (2017) 76:11–7. doi: 10.1016/j.comppsych.2017.02.010, PMID: 28384524

[24] Su CF Kuo TB Kuo JS Lai HT Chen HI. Sympathetic and parasympathetic activities evaluated by heart-rate variability in head injury of various severities. Clin Neurophysiol. (2005) 116:1273–9. doi: 10.1016/j.clinph.2005.01.010 15978489

[25] Piccirillo G Elvira S Bucca C Viola E Cacciafesta M Marigliano V. Abnormal passive head-up tilt test in subjects with symptoms of anxiety power spectral analysis study of heart rate and blood pressure. Int J Cardiol. (1997) 60:121–31. doi: 10.1016/s0167-5273(97)00088-0, PMID: 9226281

[26] Howard LM Khalifeh H. Perinatal mental health: a review of progress and challenges. World Psychiatry. (2020) 19:313–27. doi: 10.1002/wps.20769, PMID: 32931106 PMC7491613

[27] Goodman JH. Perinatal depression and infant mental health. Arch Psychiatr Nurs. (2019) 33:217–24. doi: 10.1016/j.apnu.2019.01.010, PMID: 31227073

[28] Alder J Fink N Bitzer J Hosli I Holzgreve W. Depression and anxiety during pregnancy: a risk factor for obstetric, fetal and neonatal outcome? A critical review of the literature. J Matern Fetal Neonatal Med. (2007) 20:189–209. doi: 10.1080/14767050701209560, PMID: 17437220

[29] Chung TK Lau TK Yip AS Chiu HF Lee DT. Antepartum depressive symptomatology is associated with adverse obstetric and neonatal outcomes. Psychosom Med. (2001) 63:830–4. doi: 10.1097/00006842-200109000-00017, PMID: 11573032

[30] Grote NK Bridge JA Gavin AR Melville JL Iyengar S Katon WJ. A meta-analysis of depression during pregnancy and the risk of preterm birth, low birth weight, and intrauterine growth restriction. Arch Gen Psychiatry. (2010) 67:1012–24. doi: 10.1001/archgenpsychiatry.2010.111, PMID: 20921117 PMC3025772

[31] Yonkers KA Smith MV Forray A Epperson CN Costello D Lin H. Pregnant women with posttraumatic stress disorder and risk of preterm birth. JAMA Psychiatry. (2014) 71:897–904. doi: 10.1001/jamapsychiatry.2014.558, PMID: 24920287 PMC4134929

[32] National Health Commission of the People’s Republic of China Eexplore the special service plan for depression prevention and treatment. Available online at: https://www.gov.cn/zhengce/2020-09/11/content_5542560.htm (2020).

[33] Ahlskog G. Clinical hypnosis today. Psychoanal Rev. (2018) 105:425–37. doi: 10.1521/prev.2018.105.4.425, PMID: 30063419

[34] Clark L. Temperament as a unifying basis for personality and psychopathology. J Abnorm Psychol. (2005) 114:505–21. doi: 10.1037/0021-843X.114.4.505, PMID: 16351374

[35] Landolt AS Milling LS. The efficacy of hypnosis as an intervention for labor and delivery pain: a comprehensive methodological review. Clin Psychol Rev. (2011) 31:1022–31. doi: 10.1016/j.cpr.2011.06.002, PMID: 21762655

