# Supplementary figures and images for: Effects of group hypnotic intervention on pregnant mental health and delivery mode: a retrospective analysis
Source: Front Med (Lausanne). 2025 Oct 30;12:1671398. doi: 10.3389/fmed.2025.1671398 (PMC12611927; doi:10.3389/fmed.2025.1671398)

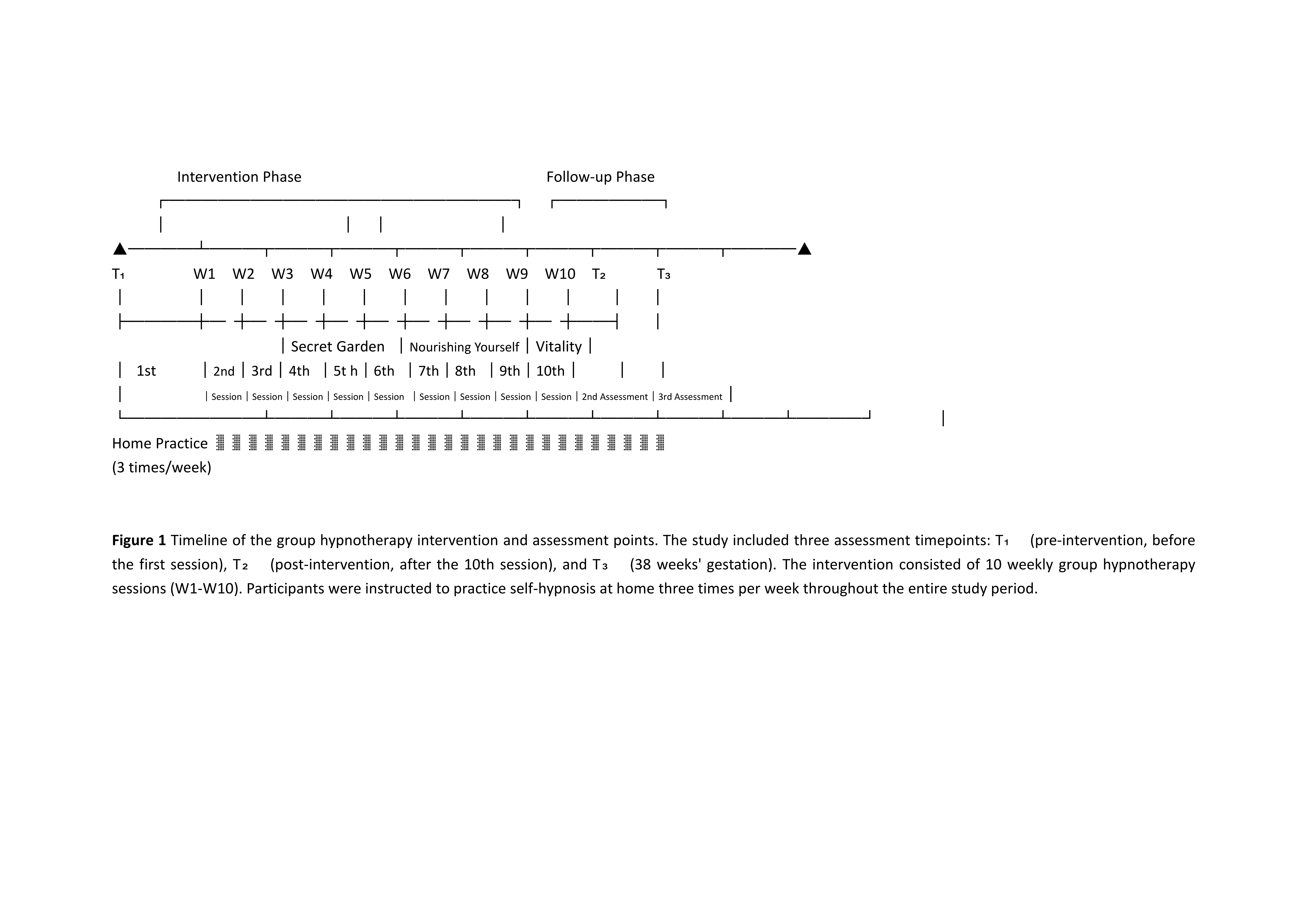

Supplement: Supplementary file 1 [file Image_1.JPEG]

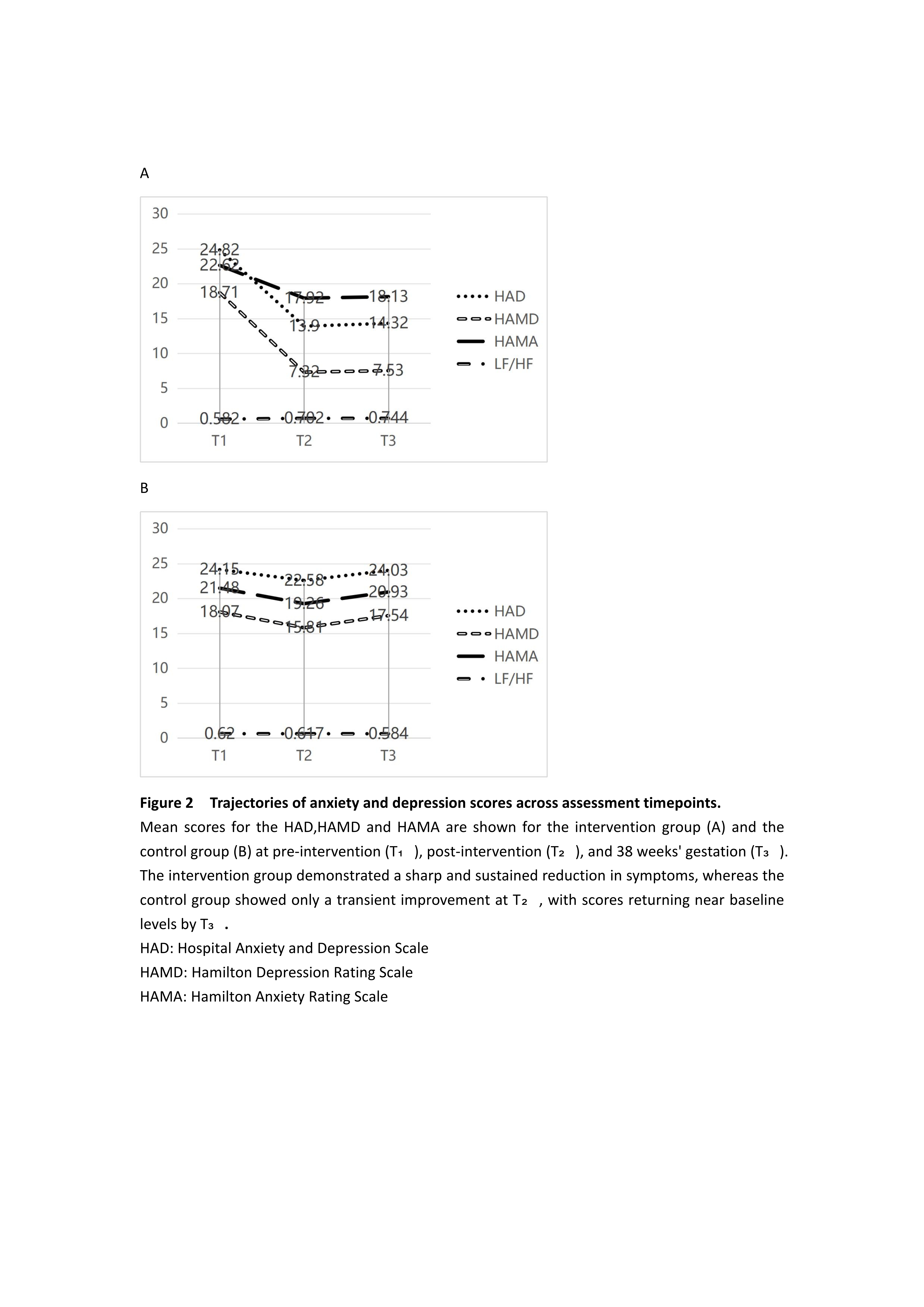

Supplement: Supplementary file 2 [file Image_2.JPEG]

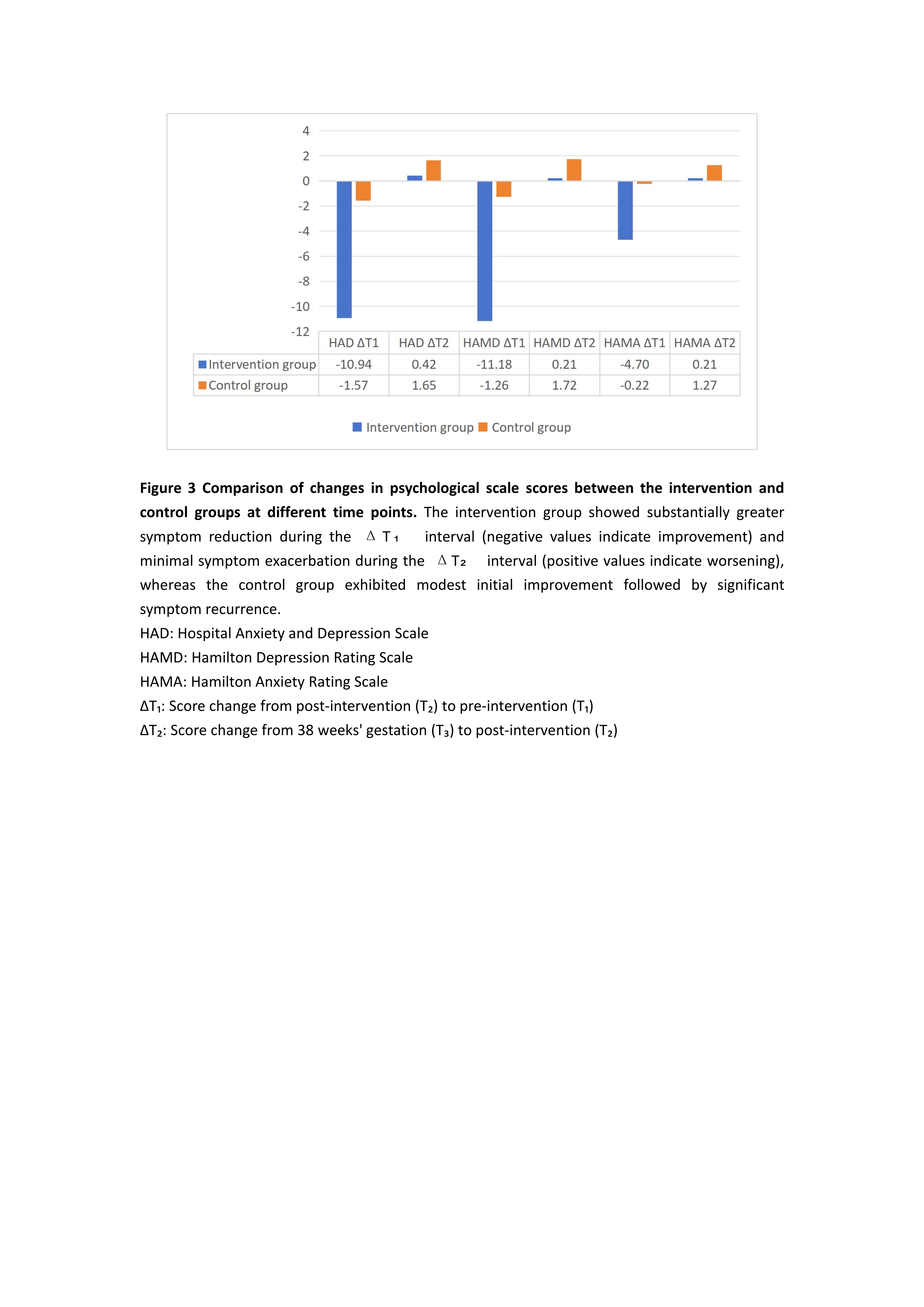

Supplement: Supplementary file 3 [file Image_3.JPEG]
